# Systematic microcarrier screening and agitated culture conditions improves human mesenchymal stem cell yield in bioreactors

**DOI:** 10.1002/biot.201400862

**Published:** 2016-02-29

**Authors:** Qasim A. Rafiq, Karen Coopman, Alvin W. Nienow, Christopher J. Hewitt

**Affiliations:** ^1^ Centre for Biological Engineering, Department of Chemical Engineering, Loughborough UniversityLeicestershireUnited Kingdom; ^2^ Wolfson School of Manufacturing and Mechanical Engineering, Loughborough UniversityLeicestershireUnited Kingdom; ^3^ Aston Medical Research Institute, School of Life and Health Sciences, Aston University, Aston TriangleBirminghamUnited Kingdom; ^4^ School of Chemical Engineering, University of Birmingham, EdgbastonBirminghamUnited Kingdom

**Keywords:** Bioreactor, Cell therapy bioprocessing, Human mesenchymal stem cell, Microcarrier, Regenerative medicine

## Abstract

Production of human mesenchymal stem cells for allogeneic cell therapies requires scalable, cost‐effective manufacturing processes. Microcarriers enable the culture of anchorage‐dependent cells in stirred‐tank bioreactors. However, no robust, transferable methodology for microcarrier selection exists, with studies providing little or no reason explaining why a microcarrier was employed. We systematically evaluated 13 microcarriers for human bone marrow‐derived MSC (hBM‐MSCs) expansion from three donors to establish a reproducible and transferable methodology for microcarrier selection. Monolayer studies demonstrated input cell line variability with respect to growth kinetics and metabolite flux. HBM‐MSC1 underwent more cumulative population doublings over three passages in comparison to hBM‐MSC2 and hBM‐MSC3. In 100 mL spinner flasks, agitated conditions were significantly better than static conditions, irrespective of donor, and relative microcarrier performance was identical where the same microcarriers outperformed others with respect to growth kinetics and metabolite flux. Relative growth kinetics between donor cells on the microcarriers were the same as the monolayer study. Plastic microcarriers were selected as the optimal microcarrier for hBM‐MSC expansion. HBM‐MSCs were successfully harvested and characterised, demonstrating hBM‐MSC immunophenotype and differentiation capacity. This approach provides a systematic method for microcarrier selection, and the findings identify potentially significant bioprocessing implications for microcarrier‐based allogeneic cell therapy manufacture.

**Abbreviations**
DMEM: Dulbecco's Modified Eagle Medium
FBS: foetal bovine serum
hMSC: human mesenchymal stem cell
hBM‐MSC: human bone marrow‐derived mesenchymal stem cell
ISCT: International Society for Cellular Therapy
PLGA: poly(lactic‐co‐glycolic acid)

## 1 Introduction

Pioneering work in the 1970s led to the isolation and identification of mesenchymal stem/stromal cells (MSCs) 1, 2; these cells are now considered to be a promising candidate for cell‐based therapies, tissue engineering and regenerative medicine applications due to their multipotency, propensity to grow in vitro, promising efficacy data, ease of isolation and immune modulatory properties 3, 4, 5, 6, 7. However, the effective transfer of human MSCs (hMSCs) into widespread clinical application will depend, to a large extent, on the development of large‐scale manufacturing platforms to produce fully functional cells at the capacity required to meet clinical demand.

**Table 1:** Properties of commercially available microcarriers

Microcarrier	Manufacturer	Diameter (µm)	Matrix	Average density	Surface coating	Surface charge	Carrier porosity	Usage in this study
**Mammalian protein‐coated microcarriers**								
Collagen	SoloHill Eng. Inc.	125–212	Polystyrene	1.02	Type I porcine collagen	None	Non‐porous	✓ (Coll)
Cultispher‐G^®^	Percell‐Biolytica	130–380	Type I porcine gelatin	1.04	None	None	Macroporous (porosity: 50% pore size: 10–30 µm)	✓ (CG)
Cytodex 3™	GE Healthcare	141–211	Dextran	1.04	Type I porcine collagen	None	Non‐porous	✓ (Cyto3)
FACT III	SoloHill Eng. Inc.	125–212	Polystyrene	1.02	Cationic Type I porcine collagen	+	Non‐porous	✓ (FACT)
SphereCol^®^	Advanced BioMatrix	125–212	Polystyrene	1.03	Type I human collagen (VitroCol^®^)	None	Non‐porous	✗
**Recombinant protein‐coated microcarriers**								
ProNectin^®^ F	SoloHill Eng. Inc.	125–212	Polystyrene	1.02	Recombinant fibronectin	None	Non‐porous	✓ (Pro‐F)
**Xeno‐free microcarriers**
Cytodex 1™	GE Healthcare	147–248	Dextran	1.03	DEAE	+	Non‐porous	✓ (Cyto1)
Cytopore 1 and 2™	GE Healthcare	200–280	Cotton cellulose	1.03	DEAE	+	Micro/Macroporous (porosity: > 90% pore size: 30 µm)	✗
Enhanced Attachment	Corning	125–212	Polystyrene	1.02	CellBIND^®^	None	Non‐porous	✓ (EA)
Glass	SoloHill Eng. Inc.	125–212	Polystyrene	1.02	High silica glass	None	Non‐porous	✗
Hillex^®^ CT	SoloHill Eng. Inc.	90–212	Polystyrene	1.12	Cationic trimethyl ammonium	+	Non‐porous	✗
Hillex^®^	SoloHill Eng. Inc.	160–180	Dextran	1.11	Cationic trimethyl ammonium	+	Non‐porous	✓ (Hillex)
MicroHex™	Nunc	Side‐length: 125 µm Thickness: 25 µm	Polystyrene	1.05	Nunclon™ surface	Not specified	Non‐porous	✓ (MHex)
Plastic	SoloHill Eng. Inc.	125–212	Polystyrene	1.02	None	None	Non‐porous	✓ (Plas)
Plastic Plus	SoloHill Eng. Inc.	125–212	Polystyrene	1.02	None	+	Non‐porous	✓ (Pplus)
PVA	Loughborough University	100–220	PVA	1.03	None	None	Non‐porous	✓ (PVA)
Synthemax II^®^	Corning	125–212	Polystyrene	1.02	Synthemax II^®^	None	Non‐porous	✓ (Sy)

Various expansion platforms have been considered for hMSCs, including traditional monolayer expansion systems such as T‐flasks and cell factories 8, 9, hollow‐fibre based bioreactor systems 10, 11 and microcarrier expansion in stirred‐tank bioreactors 12, 13, 14. For a detailed review and comparison of the advantages and disadvantages of various culture systems (and anchorage‐dependent cells in general), see recent reviews 15, 16, 17.

Microcarrier‐based bioreactor systems benefit from a significantly larger surface area to volume ratio compared to monolayer culture as well as providing the additional advantages of process control, flexible operation (i.e. batch, fed‐batch, perfusion), homogenous culture conditions via impeller agitation, readily‐accessible sampling for both medium and cell material and the ability to harvest in situ 17, 18, 19, 20, 21. It is because of this that a significant amount of research is being conducted into these systems for both hMSCs 13, 22, 23, 24, 25 as well as other anchorage‐dependent cell types 26, 27, 28.

Without going into detail regarding the hMSC‐microcarrier studies, as this is covered elsewhere 17, 19, it is worth noting that there have been many different types of microcarriers used, each with a different structure (i.e. solid bead, macroporous, enzymatically digestible), coatings (fibronectin, collagen, gelatin) and particle sizes. Despite many studies involving hMSCs and microcarrier culture investigating various aspects of the culture process, there is no unified set of culture conditions for hMSC microcarrier expansion. This is in part understandable given inevitable differences arising due to donor variation as well as the relative infancy of the research, with the earliest successful MSC microcarrier expansion being reported only in 2007 22.

The selection of the appropriate microcarrier is a fundamental component of microcarrier/stirred‐tank bioreactor cell expansion. From a cell therapy processing perspective, it is expected that once selected, the microcarrier of choice will need to remain the same throughout, particularly during the later stages of clinical development (Phase II and Phase III) at which point the process is ‘locked in’. Any change at this point is likely to constitute a major change in the manufacturing process, requiring significant comparability testing and repeats of clinical trials. Therefore, it is of paramount importance that the optimal microcarrier is selected from the outset based on a stringent selection methodology.

Much of the work conducted in the literature does not specify why or how a specific microcarrier was selected for expansion, nor is it clear if any strategy or procedure was put in place to identify the optimal microcarrier, or indeed outline the criteria for what the ‘ideal’ microcarrier should be. Given the significance this has on the entire cell therapy production process, it is surprising that no in‐depth study has been performed comparing the various commercially available microcarriers. Schop and colleagues 23 conducted a microcarrier screen comparing nine different commercially available microcarriers (Cytodex‐1 and ‐3, and all SoloHill microcarriers; Table 1), yet the study was limited to identifying the extent of cell attachment over an 18 h period as determined by the Alamar Blue assay. They found that Cytodex‐1 allowed for the greatest seeding efficiency, and as such, conducted further research with this microcarrier only. However this study did not take into account hMSC growth kinetics after 18 h, nor did it take into account other key issues such as the harvesting of cells from the microcarriers.

Kehoe and colleagues performed a similar study which included an investigation of cell recoverability and found that whilst Cytodex‐1 appeared to best facilitate cell growth, it was significantly lower with respect to cell recoverability in comparison with the SoloHill Collagen microcarrier 24. Whilst the study does provide some interesting insights, the study appears truncated and has not been peer‐reviewed. Moreover, the study used only one hMSC line and provides little detail as to how cell growth for the microcarrier screen was determined. Whilst there have been attempts to select an appropriate microcarrier, it is clear there is no robust, rigorous, transferable process for microcarrier selection.

This study systematically evaluates microcarriers for hBM‐MSCs from three different donors, provides a set of key criteria which define the ‘ideal’ microcarrier and provides a reproducible and transferable methodology for selecting an appropriate microcarrier for hBM‐MSC culture.

## 2 Materials and methods

### 2.1 hBM‐MSC monolayer expansion

Human MSCs from three different healthy donors were isolated from bone‐marrow aspirate obtained by Lonza (Lonza, Walkersville, USA) after the donor provided informed consent. The local Ethical Committee approved the use of the samples for research. The donors were selected on the basis of different ages, genders and ethnicities which are as follows: hBM‐MSC1 (20 years old, male, black), hBM‐MSC2 (19 years old, female, black), hBM‐MSC3 (24 years old, male, caucasian). The hBM‐MSCs were isolated on the basis of plastic adherence and cryopreserved at passage 1 at a density of 2 × 10^6^ cells/mL in 10% v/v dimethyl sulphoxide (DMSO) (Sigma Aldrich, UK) and 90% foetal bovine serum (FBS; Hyclone, Lot# RUF35869). The cells were expanded until passage 3 at which point experimental work began. Cells were cultured in complete growth medium, which consisted of Dulbecco's Modified Eagle Medium (DMEM; Lonza, UK) supplemented with 10% v/v foetal bovine serum (FBS; Hyclone, Lot# RUF35869) and 2 mM ultraglutamine (Lonza, UK).

Monolayer expansion of hBM‐MSCs involved seeding T‐flasks or plates at a density of 5000 cells/cm^2^ and then placed in a humidified CO_2_ controlled incubator at 37°C which had air supplemented with 5% CO_2_. A complete medium exchange was performed after 72 h of culture and cells were passaged upon reaching confluency (day 6 of culture). Passaging involved washing the hBM‐MSC monolayer with Ca^2+^ and Mg^2+^ free phosphate buffered saline (PBS; Lonza, UK) followed by an incubation step for 5 min with trypsin (0.25%)/EDTA solution (Lonza, UK) to dissociate the cells from the surface of the tissue culture plastic. Complete growth medium was added to inactivate the trypsin at the three‐fold volume of the trypsin solution used for cell detachment. The cell suspension was then centrifuged at 220 g for 5 min at room temperature, the supernatant discarded and the remaining pellet re‐suspended in an appropriate volume of culture medium. The viable cell number was determined by using the Nucleocounter NC‐3000 image cytometer (Chemometec, Denmark) following the manufacturer's instructions.

### 2.2 Microcarrier culture

All microcarrier cultures were performed in vessels or plates designed to avoid attachment of the cells to the plate or vessel surface. This was achieved in multi‐well plates by using ultra‐low attachment plates (Corning, UK), whilst spinner flask vessels were coated in Sigmacote (Sigma Aldrich, UK). Sigmacote was used to treat glass vessels to siliconize the surface thereby preventing cell attachment to the vessel surface. Sigmacote was applied to the entire vessel surface area and aspirated. Vessels were left overnight to dry in a fume hood and rinsed with distilled water after 24 h and autoclaved prior to use.

All microcarriers used in this investigation (Table 1) were prepared according to the manufacturer's instructions. For all microcarrier cultures, after initial sterilization by autoclaving, the microcarriers were left in complete growth medium for at least 1 h in a humidified incubator at 37°C and 5% CO_2_ to allow for the conditioning of the microcarriers and thereby aiding cell attachment. After this period, the conditioning medium was aspirated, taking care to avoid aspirating microcarriers, and fresh, pre‐warmed growth medium was added to the volume. Cells were then inoculated at a seeding density of 6000 cells/cm^2^, greater than the 5000 cells/cm^2^ seeding density for monolayer culture. This was done so as to take into account cell loss due to the lack of attachment to a microcarrier.

### 2.3 Spinner flask configuration

100 mL BellCo spinner flasks (BellCo, US), were used for all spinner flask experiments, with a 100 mL working volume and a vessel diameter (*T*) of 60 mm. Agitation was achieved by a magnetic horizontal stir bar and a vertical paddle (diameter, *D* = 50 mm). Spinner flasks were set up on a Bell‐Ennium^™^ Compact five position magnetic stirrer platform (BellCo, US), maintained in a 37°C, humidified incubator which had air supplemented with 5% CO_2_.

In spinner flask cultures, after microcarrier inoculation, the vessels were warmed to 37°C but not agitated for a period of 24 h, this was done to allow the cells to settle and attach to the microcarriers. At all times whilst in the incubator, a side‐arm of the spinner flask was slightly loosened (half a turn of the cap) to allow for sufficient gas exchange. After the initial settling period, the vessels were agitated; the spinner flasks were placed onto the Bell‐Ennium^™^ Compact five position magnetic stirrer platform which allowed for the controlled agitation of the spinner flask via a magnetic stirrer.

The agitation speed for the spinner flasks was set to *N*_JS_ (the impeller speed at which the microcarriers are just suspended 18), which was found to be 30 rpm for the spinner flasks on the Bell‐Ennium^™^ platform. For a detailed discussion as to why this speed was selected, the reader is directed to our liter‐scale stirred‐tank expansion study 14.

Cell attachment in the spinner flask cultures was determined by taking supernatant samples one hour after inoculation to ascertain the numbers of unattached cells. Daily 1 mL samples were also taken throughout culture for analysis.

### 2.4 Determination of metabolite concentration and metabolic activity

To determine the metabolite concentration in the hBM‐MSC cultures, 1 mL of spent medium was aseptically transferred to an Eppendorf tube and then run on the Bioprofile FLEX bioanalyser for analysis of glucose in mmol/L, lactate in mmol/L, and ammonium in mmol/L.

The specific metabolite consumption/production rate was determined by

(1) $$\begin{array}{l} \mathrm{Specific}\;\mathrm{metabolite}\;\mathrm{flux},\;q_{\mathrm{met}}=\\ \left(\frac{\mu }{c_{x}(\text{0})}\right)\cdot \left(\frac{c_{\mathrm{met}}(t)-c_{\mathrm{met}}(\text{0})}{e^{\mathrm{\mu t}}-\text{1}}\right) \end{array}$$

where *q*_met_= specific metabolite consumption rate, µ = specific growth rate (h^−1^), *C*_met(t)_ and *C*_met(0)_ = concentration of metabolite at the start and end of exponential growth phase respectively, *C*_x(0)_ = cell number at the end of exponential growth phase and *t* = time (h).

The WST‐1 assay (Roche, UK) was used to determine the metabolic activity of viable cells; a colorimetric test, the principle of the assay is the reduction of WST‐1 by viable cells, producing a soluble and visible formazan salt which can be measured using an ELx800 plate reader (Invitrogen, UK).

### 2.5 Flow cytometry

Immunophenotypic analysis of the hBM‐MSCs was determined by flow cytometry. This was performed using the Quanta SC flow cytometer with excitation at 488 nm. Cells were prepared for analysis following enzymatic harvesting from the growth surface and centrifuging at 300 ×*g*. The supernatant was discarded and the cells were resuspended in flow cytometry stain buffer (R&D Systems, UK). A panel of mouse anti‐human monoclonal antibodies was used to target cell‐surface receptors and was prepared in accordance with the manufacturer's instructions. The antibodies were based on the panel recommended by the International Society for Cellular Therapy (ISCT) 29 and included CD73‐PE, CD90‐PE CD105‐PerCP, CD14 FITC, CD19 FITC CD34‐PE‐Cy7 CD45‐PE‐Cy5, and HLA‐DR‐FITC (R&D Systems, UK). Cells were incubated with the antibody in the dark at room temperature for 30 min. Associated isotype controls were also prepared for all experimental conditions. A minimum of 10^3 ^events were recorded for each sample and the data were analysed using FlowJo computer software (Treestar Inc, USA).

### 2.6 Differentiation of hBM‐MSCs

The multi‐lineage potential of the cells was ascertained by inducing the samples post‐harvest with the StemPro Adipogenesis kit, StemPro Chondrogenesis kit and StemPro Osteogenesis kit (Life Technologies, UK). The media were prepared and used as per the manufacturer's instructions and the differentiation studies were performed as described in 30.

### 2.7 Harvesting of hBM‐MSCs

A detailed overview of the harvesting method and theoretical principles are provided in 20, briefly however, the harvesting procedure began by allowing the microcarriers to settle after which the medium was aspirated. The microcarriers were then washed with 100 mL of Ca^2+^ and Mg^2+^ free PBS. The washing procedure involved placing the spinner flasks on a spinner platform and agitating at *N*_JS_ (30 rpm) for 5 min. The microcarriers were again allowed to settle and the excess PBS was aspirated. The wash procedure was repeated after which 60 mL trypsin (0.25%)/EDTA was added to the spinner flasks and the spinner flasks were placed in a humidified CO_2_ controlled incubator for 7 min. During this incubation period, the vessels were agitated at 150 rpm to aid detachment (please refer to 20 for further details and theoretical principles). After incubation, the cells were quenched with 70 mL growth medium and vacuum filtered using a Steriflip^®^ 60 µm filtration unit (Millipore, UK). The cell suspension was then centrifuged at 220 ×*g* for 5 min and resuspended in growth medium.

### 2.8 Statistical analysis

Statistical significance was determined by analysis of two groups of data using the Mann–Whitney *U* test and significance was determined at *p* < 0.05.

## 3 Results

### 3.1 Monolayer expansion demonstrates variation in growth kinetics between three different hBM‐MSC lines

Prior to microcarrier expansion, the monolayer proliferative capacity of the hBM‐MSCs from the three donors (hBM‐MSC1, hBM‐MSC2, hBM‐MSC3) were compared. The cells were expanded on tissue culture plastic for three passages from passage 3 to passage 5 (the cells' actual passage number), hereafter referred to as passage 1 to passage 3 (the experimental passage number).

**Figure 1:**
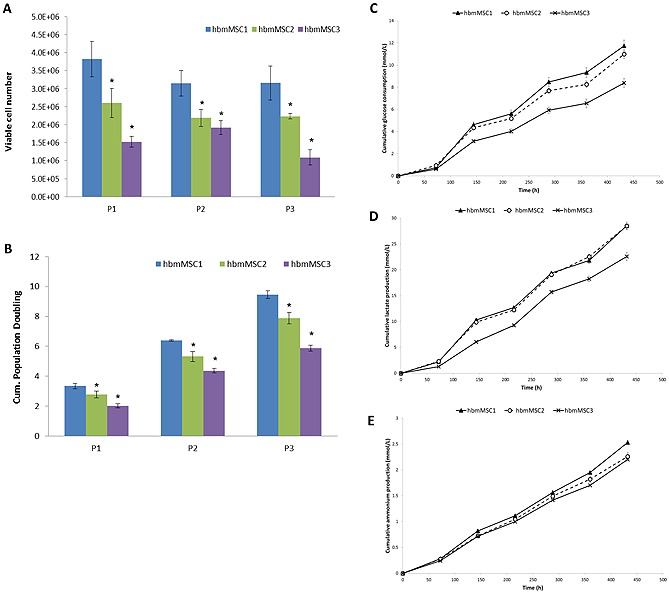
Monolayer culture growth kinetics and metabolite concentrations for three donor hBM‐MSCs across three passages. Viable cell number (**A**) and cumulative population doublings (**B**) data for hBM‐MSC1, hBM‐MSC2 and hBM‐MSC3 when cultured in monolayer for three consecutive passages. Cumulative glucose consumption (**C**), lactate production (**D**) and ammonium production (**E**) for each donor hBM‐MSCs across the three passages. Data presented as mean ± standard deviation (SD) (*n* = 4). Significant differences of viable cell numbers and cumulative population doublings between hBM‐MSC2 and hBM‐MSC3 were noted with *p* < 0.05 (*) in comparison to hBM‐MSC1.

Significant differences were observed with respect to the viable cell number between hBM‐MSC1 and hBM‐MSC2 (*p* < 0.01) and hBM‐MSC1 and hBM‐MSC3 (*p* < 0.005) for passages 1 and 2, and the difference is exacerbated by passage 3 (Fig. 1A). The same trend is reflected in the cumulative population doublings, where, after three passages, hBM‐MSC1 reaches about ten cumulative population doublings, hBM‐MSC2 reaches about eight and hBM‐MSC reaches about six (Fig. 1B). Similarly, hBM‐MSC1 consistently yields a greater number of cells across the three passages (∼3.0 to 4.0 × 10^6^ cells) in comparison to hBM‐MSC2 (2.0 to 2.5 × 10^6^ cells) and hBM‐MSC3 (1.0 to 2.0 × 10^6^ cells) (Fig. 1A). In addition to this, the metabolite data are indicative of greater cell proliferation for hBM‐MSC1, where, after three passages, the cumulative glucose consumption (Fig. 1C) was found to be the highest for hBM‐MSC1 (∼12.0 mmol/L), followed by hBM‐MSC2 (∼11.0 mmol/L) and hBM‐MSC3 (∼8.5 mmol/L). The cumulative lactate production (Fig. 1D) for hBM‐MSC1 and hBM‐MSC2 were almost identical (28.53 mmol/L for hBM‐MSC1 and 28.50 for hBM‐MSC2) whilst for hBM‐MSC3 this was demonstrably lower (∼22.5 mmol/L). The cumulative ammonium production (Fig. 1E) followed a similar trend, with hBM‐MSC1 producing the most ammonium (2.53 mmol/L), hBM‐MSC2 producing 2.25 mmol/L and hBM‐MSC3 producing hBM‐MSC3 2.2 mmol/L.

The data illustrate the innate biological variation between the three donor hBM‐MSCs, with hBM‐MSC1 performing better with respect to cell growth than hBM‐MSC2, and hBM‐MSC2 performing better than hBM‐MSC3. This variation is reflected in the nutrient consumption and metabolite production, whereby more glucose is consumed and more lactate and ammonium produced in hBM‐MSC1 cultures in comparison to hBM‐MSC2 and hBM‐MSC3. It has been found that in general, cell proliferation correlates with nutrient consumption and production of potentially detrimental by‐products such as lactate and ammonium 31, which is reflected by the findings here. All experimental parameters (i.e. time in culture, medium, tissue culture flask, operator etc.) between the monolayer cultures were standardized, as such, the variation in growth kinetics is likely to have arisen due to donor variation.

Based on the findings of the monolayer study, the microcarrier screening studies were performed with all three donor hBM‐MSCs to ascertain whether the donor cells would demonstrate better cell proliferation on certain microcarriers, or whether a separate microcarrier for each donor would be needed. Moreover, all donor cells were used to identify whether the trend observed in the monolayer conditions (i.e. hBM‐MSC1 performing better with respect to cell proliferation than hBM‐MSC2, and hBM‐MSC2 better than hBM‐MSC3) was also observed when culturing the cells on microcarriers.

### 3.2 Screening in ultra‐low microwell attachment plates provides a robust scale‐down model for microcarrier selection

Prior to performing the microcarrier screen, the selection criteria for the ‘ideal’ microcarrier for hBM‐MSC culture were established. This focused on three important attributes:

1. Level of cell proliferation as determined by viable cell number, or appropriate orthogonal measurements (i.e. WST‐1 and metabolite analysis) relative to the other microcarriers.
2. Amenability for xeno‐free processing.
3. Ability to effectively harvest the cells from the microcarriers without any detrimental effect on the cells' immunophenotype or differentiation capacity.

A range of commercially available microcarriers (Table 1) were screened initially in static microwell plates to identify an optimal microcarrier for the culture of hBM‐MSCs. Duplicate ultra‐low attachment plates were prepared with each plate containing 13 different microcarriers and a control well not containing any microcarrier at all (No MC). Microcarriers used were selected on the basis of: successful prior use for hBM‐MSCs expansion (e.g. Cytodex‐1, Hillex II and Cultispher G), successful culture of other cells types (e.g. in vaccine production using CHO) and microcarrier physical and chemical properties.

**Figure 2:**
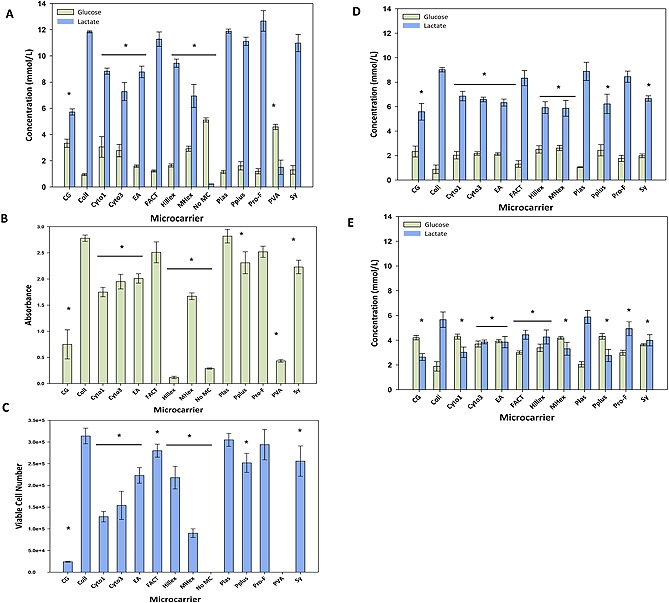
Comparison of microcarriers for hBM‐MSC culture in static microwell plates. For hBM‐MSC1 expansion, the glucose consumption and lactate production (**A**), WST‐1 absorbance (**B**) and viable cell number (**C**) for each microcarrier is given. Glucose consumption and lactate production for hBM‐MSC2 (**D**) and glucose consumption and lactate production for hBM‐MSC3 (**E**). The control condition (No MC) contained no microcarriers and no cell attachment was expected as ultra‐low attachment plates were used. Data is presented as mean ± standard deviation (SD) (*n* = 8). Significant differences in values were noted with *p* < 0.05 (*) in comparison to the highest respective value for each assay.

Since obtaining viable cell number (Fig. 2C) on microcarriers can be difficult to achieve in practise and prone to error, glucose and lactate concentrations (Fig. 2A), WST‐1 assay absorbance readings (Fig. 2B) of hBM‐MSCs grown on different microcarriers were also measured, with the former generally correlating well with cell growth 32. In general, it was found that the cell proliferation analyses resulted in reproducible data as indicated by the tight error bars, with the data from each analytical technique corroborating the findings of the other analyses. As expected the largest error bars were obtained for the viable cell number data (Fig. 2C).

The data from the control well for hBM‐MSC1 demonstrates that cells were unable to attach to the ultra‐low attachment surface and proliferate (Fig. 2A–2C and Supporting information, Fig. S1I). This data was similar to that found for the PVA microcarriers (Supporting information, Fig. S1M) where the lactate concentration never surpassed the glucose concentration during the course of the culture, with the glucose concentration being relatively high at ∼4.5 mmol/L after 144 h and the corresponding lactate concentration being relatively low (∼1.8 mmol/L). Likewise, the WST‐1 absorbance reading is similar to the control condition with a value of ∼0.45 at 144 h, whilst no viable cells were detected after 144 h. It was therefore decided that these microcarriers would not be considered for further investigations. Of the remaining microcarriers, Cultispher‐G was also considered to have performed poorly since it had a high glucose concentration (∼3.8 mmol/L), a low lactate concentration (∼6 mmol/L) and low WST‐1 absorbance reading (∼0.75) at 144 h. However, unlike with PVA, it did allow for the attachment and proliferation of hBM‐MSCs, albeit at a very low value of ∼2.5 × 10^4^ cells after 144 h.

The Hillex microcarriers demonstrated cell proliferation as identified by the change in metabolite concentrations and increasing viable cell number, yielding ∼2.25 × 10^5^ cells. Nevertheless the WST‐1 absorbance readings were the lowest throughout the culture and did not change at 144 h (Fig. 2B). This anomalous reading however is not unexpected given that the Hillex microcarriers absorb the phenol red indicator present in the DMEM (Supporting information, Fig. S1M). As a result, it was not possible to obtain accurate WST‐1 absorbance readings and the WST‐1 data for Hillex were disregarded. In general, it was found that for hBM‐MSC1, the two Corning microcarriers (Enhanced Attachment and Synthemax II, Supporting information, Fig. S1E and S1N, respectively) and SoloHill microcarriers (Collagen, FACT, Hillex, Plastic, Plastic Plus and ProNectin‐F, Supporting information, Fig. S1B, S1F, S1G, S1J, S1K and S1L, respectively) were the most effective for cell proliferation, with respect to metabolite concentrations, WST‐1 absorbance readings and viable cell number.

To ascertain whether the relative performance of the microcarriers would differ with different donor cells, the investigation was repeated using hBM‐MSC2 and hBM‐MSC3 with the exception that the PVA microcarriers were excluded based on previous poor performance. Figures 2D and 2E provide the metabolite concentrations for hBM‐MSC2 and hBM‐MSC3 respectively, which illustrate that whilst the effect may not be as pronounced, the trend with respect to microcarrier performance is the same, with the SoloHill microcarriers (in particular Collagen, FACT, Plastic and ProNectin‐F) generally consuming more glucose and producing more lactate after 144 h for both hBM‐MSC2 and hBM‐MSC3.

Interestingly, these data correlate well with the monolayer metabolite profiles (Fig. 2A–2C) for hBM‐MSC1, hBM‐MSC2 and hBM‐MSC3, with hBM‐MSC1 cultures generally consuming more glucose and producing more lactate in comparison to hBM‐MSC2, and likewise hBM‐MSC2 more than that of hBM‐MSC3. Given that the nutrient/metabolite concentrations, as indicated in the earlier part of this paper, are a relative measure for cell growth, this would suggest hBM‐MSC1 cultures yielded more cells than hBM‐MSC2, and likewise hBM‐MSC2 more than hBM‐MSC3, which mirrors the findings of the monolayer cultures (Fig. 1).

### 3.3 Agitated culture conditions significantly improve hMSC yield for all hBM‐MSC donor lines

Whilst the studies conducted in the ultra‐low attachment microwell plates provided an indication as to the performance of the microcarriers, it was important to conduct spinner flask based experiments for microcarrier comparison, as this would be more representative of how the microcarriers would be employed for hBM‐MSC growth in practise. In addition to this, static vs. agitated conditions were compared, whereby the same concentration of microcarriers and cells were inoculated, the only difference being that one set of spinner flasks was agitated, whilst the other set was not. HBM‐MSCs from the three donors were cultured on all microcarriers used in Section 3.2 with the exception of the PVA microcarriers which were discontinued for the reasons stated previously.

**Figure 3:**
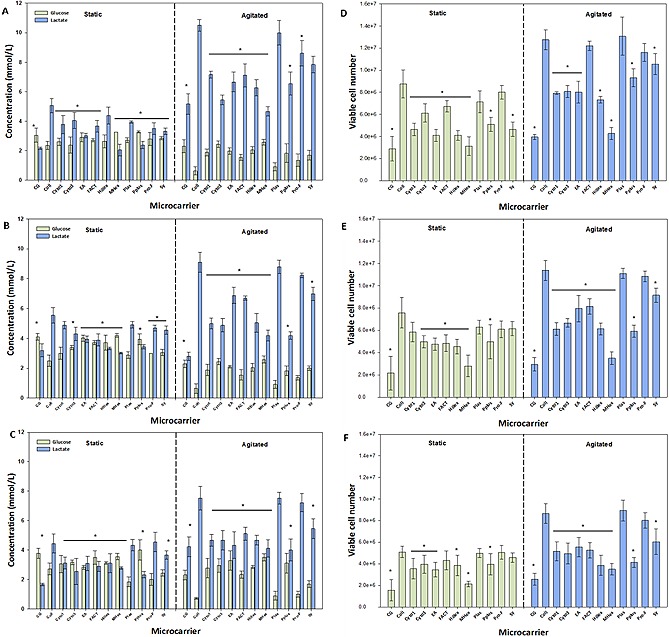
Comparison of static and agitated conditions for hBM‐MSC microcarrier culture, and a comparison of different microcarriers in 100 mL spinner flasks. Glucose consumption and lactate production for hBM‐MSC1 (**A**), hBM‐MSC2 (**B**) and hBM‐MSC3 (**C**). Viable cell number for hBM‐MSC1 (**D**), hBM‐MSC2 (**E**) and hBM‐MSC3 (**F**). Data is presented as mean ± standard deviation (SD) (*n* = 4). Significant differences in values were noted with *p* < 0.05 (*) in comparison to the highest respective value.

Of particular note is the significant difference between the static and agitated culture conditions (Fig. 3). At 144 h, under static culture conditions, the metabolite concentrations for each of the different microcarriers for all three donor hBM‐MSCs were similar, with glucose concentrations ranging between ∼2 and 3 mmol/L, and lactate between ∼2 and 5 mmol/L, demonstrating little difference between the microcarriers with respect to metabolic activity under static conditions (Fig. 3A–3C). This is reflected by the viable cell number data (Fig. 3D–3F), where similar cell numbers were obtained between the microcarriers, ranging from ∼4 to 9 × 10^6 ^cells across the three different hBM‐MSC lines. However under agitated conditions at 144 h, the glucose concentrations were generally below 2.5 mmol/L for most microcarriers and greater than 5 mmol/L with respect to lactate, with both Plastic and Collagen resulting in the lowest glucose concentrations and highest lactate concentrations (Fig. 3A–3C). Similarly, the viable cell numbers were generally >8 × 10^6^ cells, with both Plastic and Collagen resulting in the greatest number of viable cells in the agitated conditions for all three hBM‐MSCs (Fig. 3D–3F).

In all cases for the static and agitated spinner flask cultures, the percentage of initial cell adhesion was found to be ∼55% based on identification of non‐attached cells from 1 mL supernatant samples obtained 1 h post‐seeding. The percentage of recovered cells, in all cases, was found to be >95% as determined by pre‐harvest samples and post‐harvest recovery. Unfortunately, it was not possible to quantify the attachment efficiency for the micro‐wells because of the limited sample volume available in them, i.e. taking a 1 mL supernatant sample as required for measurement from a 2 mL culture volume would make the experiment valueless.

Although the respective glucose and lactate concentrations values are lower in the static spinner flask compared to the static micro‐well plates (Fig. 2A and 3A), importantly, the general trend of microcarrier performance for the donor hBM‐MSCs is similar, with the same set of microcarriers (Collagen, Plastic, ProNectin‐F) performing better with respect to proliferation (Fig. 2 and 3).

It is likely this disparity in the concentration values between the static spinner flasks and the micro‐well plates is due to inferior attachment in the former resulting in delayed proliferation. Although the concentration of microcarriers was proportional to the volume of culture medium, the two culture platforms had different geometries and sizes. As a result, the microcarriers in the micro‐well plate covered a greater proportion of the base than in the spinner flask, to which the cells would fall under the action of gravity, thus resulting in a better attachment. In addition, the distance that the cells have to settle in the micro‐wells is less than in the spinner flask. As explained above, it was not possible to quantify the attachment efficiency for the micro‐wells.

**Figure 4:**
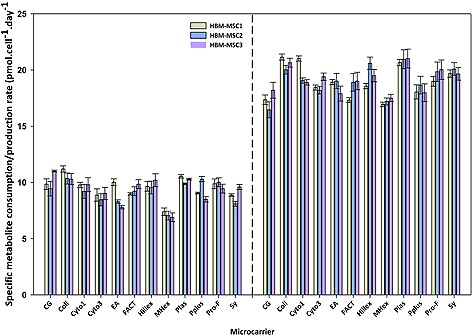
Specific glucose consumption and lactate production rates for each microcarrier and each hBM‐MSC lines in the agitated spinner flasks. Data is presented as mean ± standard deviation (SD) (*n* = 4).

In the case of the agitated spinner flasks however, once agitation is initiated, cell proliferative activity improves as a result of improved mass transfer and surface area availability (as the entire surface area of the microcarrier is now exposed rather than just the top layer as it the case when microcarriers are left static).

The specific glucose consumption and specific lactate production rates were determined for each microcarrier and each hBM‐MSC line under agitated conditions (Fig. 4). Unlike the glucose and lactate concentrations and viable cell number, it was not possible to differentiate significantly between the microcarriers on the basis of the specific metabolite flux. Generally, the specific glucose consumption rate was found to be ∼9 to 10 pmol/cell/day and the specific lactate production rate ranged between 17 and 20 pmol/cell/day. There were notable exceptions, for example, the specific glucose consumption rates for MicroHex and Cultispher‐G were consistently at ∼7 pmol/cell/day for each hBM‐MSC, which is believed to be related predominantly to the proliferative rate in this instance. A similar trend is found with the specific lactate production rate where MicroHex and Cultispher‐G have the lowest lactate production rates.

Another key finding from this study was that for all three donor hBM‐MSCs, the relative microcarrier performance was equivalent, with the same set of microcarriers performing better with respect to nutrient/metabolite consumption and production (e.g. Collagen, Plastic and ProNectin‐F), whilst the microcarriers that did not perform well with one donor hBM‐MSC generally tended not to perform well with the other donor hBM‐MSCs (e.g. Cultispher‐G, Cytodex‐3 and MicroHex). Moreover, the relative performance of the microcarriers mirrored the findings of the microwell plate study and similarly, the hBM‐MSC1 cultures outperform the hBM‐MSC2 and hBM‐MSC3 cultures with respect to the lactate production, glucose consumption and by extension, the cell growth.

Based on the findings of the screening studies, and the selection criteria outlined previously, it was decided that the SoloHill Plastic microcarrier was the most suitable given the propensity of the hBM‐MSCs to proliferate on the microcarrier and the fact that these microcarriers (unlike SoloHill Collagen) were amenable for xeno‐free processing as they are free from any animal‐derived components. The final aspect of the selection criteria was to determine whether the cells could be harvested from the Plastic microcarriers without adversely affecting their differentiation capacity or immunophenotype.

### 3.4 hBM‐MSCs retain key identity and functionality markers after microcarrier harvest

Spinner flask cultures with cells growing on Plastic microcarriers were harvested as described previously (Section 2.7). This involved a combination of incubating the cells with a dissociation reagent (in this case trypsin) and increasing the agitation speed five‐fold (in comparison to the agitation speed used for culturing, *N*_JS_). Once detached, the cells were separated from the microcarriers by filtration and harvested cells were characterized with respect to their differentiation capacity (Supporting information, Fig. S2A–C) and immunophenotype (Supporting information, Fig. S2D). It was found the cells were able to retain their ability to differentiate toward the osteogenic (Supporting information, Fig. S2A), adipogenic (Supporting information, Fig. S2B) and chondrogenic (Supporting information, Fig. S2C) lineages as determined by the Von Kossa/Alkaline Phosphatase, Oil Red O and Alcian Blue staining respectively. Similarly, the cells were analysed prior to spinner flask inoculation and post‐spinner flask harvest by flow cytometry for four positive markers (CD29, CD73, CD90 and CD105) and five negative markers (CD14, CD19, CD34, CD45, HLA‐DR), and in both cases, the cell expression was >95% for the positive markers and <2% for the negative markers. These assays demonstrate the harvested cells were in accordance with the ISCT criteria for hMSCs 29 and that the harvest process had little or no apparent effect on the cells. This fulfilled the final criterion for successful microcarrier selection that we had outlined, i.e. the ability to effectively harvest the hBM‐MSCs from the microcarrier.

The harvesting of the cells with the recently developed enzymatic‐agitation protocol from the microcarrier with best perceived characteristics (xeno‐free structure and good growth) was also successful. This result is also very encouraging because harvesting is an essential part of the production process for allogeneic therapies. Nevertheless, more work is required to establish the universality of this method and such work is in progress.

## 4 Discussion

### 4.1 Robust screening is required to select the optimal microcarrier for cell expansion

The selection of the most appropriate microcarrier is a critical component of any microcarrier‐based cell therapy production process, yet there is little in the way of systematically evaluating microcarriers, or what the criteria for selecting a microcarrier should be.

As mentioned previously Schop et al. 23 and Kehoe et al. 24 have undertaken a microcarrier screen, yet both studies only investigated the growth of one hBM‐MSC line and in the case of Schop et al. study, the assessment was based on cell attachment and proliferation over an 18‐hour period 23. Hupfeld et al. state that their selection of Cytodex‐1 for the culture of umbilical cord and amniotic membrane hMSCs was based on a microcarrier screen of six microcarriers and was based on microscopic observation, metabolic activity and expansion ratios 33. However the data are not shown nor referenced; nevertheless, they report that Cytodex‐1 was selected on the basis of expansion, which is different to what has been found here 33. Whilst it is not possible to compare the data directly, it is likely that differences found between these studies to be predominantly related to the source of hMSCs used (umbilical cord and amniotic membrane).

Li et al. (2015) selected Cultispher‐G due to the microcarrier'smacroporous and biodegradable properties for the culture of human hair follicle‐derived MSCs 34. Unlike the findings here, they report successful growth of the human follicle‐derived MSCs on Cultispher‐G. However they do not report the growth of the follicle‐derived MSCs on other microcarriers 34. The study we have conducted here would allow for a swift and robust assessment of multiple microcarriers simultaneously and enable researchers and cell therapy developers to identify the optimal microcarrier with respect to proliferation.

The microcarrier screening investigations demonstrated that whilst hBM‐MSC growth was observed on all but one of the microcarriers, some were clearly more amenable for hBM‐MSC growth than others. This was also found by Goh et al. (2013) who compared the proliferative capacity of four microcarriers (Plastic, Cytodex‐1, Cytodex‐3 and Cultispher G) for the expansion of human foetal MSCs 35. They also found that Cultispher‐G was poorest performing microcarrier investigated with a significant difference in cell density in comparison to the other microcarriers. The authors reported that Plastic and Cytodex‐3 were the joint best performing microcarriers with an overall cell density of 6.8 × 10^5^ cells/mL after 12 days of culture whilst Cytodex‐1 resulted in a density of 6.5 × 10^5^ cells/mL. The authors selected Cytodex‐3 as their microcarrier of choice due to the concern that the Plastic microcarriers resulted in a greater level of aggregation which was suggested to result in a reduction in osteogenic potential.

Similarly, work conducted by Dos Santos et al. sought to, and successfully demonstrated that the SoloHill Plastic microcarriers were equally as efficient for hBM‐MSC growth as the Cultispher‐S microcarriers that were used by the group previously 36. The reason for the change in microcarrier was due to the animal‐derived gelatin component on the Cultispher‐S microcarrier 36.

Cultispher‐G, oft‐cited in the literature 25, 37, 38, was one of the poorest performing microcarriers in both the microwell and spinner flask studies, highlighting that it is important to consider a range of microcarriers before establishing a process based on a specific microcarrier. MicroHex (hexagonally‐flat shaped with a thickness of only 25 µm), is a microcarrier whose unique selling point is that the carrier is made from the same material as Nunc's successful tissue culture plasticware (T‐flasks and multi‐well plates). Yet, surprisingly, it too did not perform well in the screening studies. MicroHex was also part of the microcarrier screen employed by Hupfeld et al. (2014) 33. However, they do not report the relative performance of the microcarriers. Given that hBM‐MSCs readily attach and proliferate on the same material when cultured in a T‐flask, one would have assumed that this would be one of the best microcarriers for hBM‐MSCs. However, as this was not the case here, it suggests that other physical properties of the microcarrier such as the shape, size and density and not only the material of construction are also likely to play an important role for successful cell growth.

Another notable finding from these studies is the wide difference in hBM‐MSC growth on the different microcarriers. It may have been assumed that as a number of these microcarriers have been employed for the successful growth of hBM‐MSCs 17, the performance of these microcarriers with respect to cell growth would be similar. Yet the data in Fig. 2, 3, 4, in addition to similar studies reported in the literature 23, 24, 35 would suggest otherwise and again highlight the fundamental importance of microcarrier selection as part of the hBM‐MSC‐microcarrier optimization process. With such a range of microcarrier performance, it may be argued that the primary consideration for hBM‐MSC microcarrier culture should be the type of microcarrier employed.

With respect to the metabolic performance of the hBM‐MSCs, the trends between cell lines were similar for each microcarrier. The specific glucose consumption and lactate production values found in this study (Fig. 4) are generally in line with those reported elsewhere. Dos Santos et al. reported specific glucose consumption values ranging between 5 and 15 pmol/cell/day, averaging at ∼10 pmol/cell/day and specific lactate production values ranging between 20 and 25 pmol/cell/day 36. Similar findings were presented by Schop et al. and appear to be indicative of hBM‐MSC microcarrier culture 23. Importantly, neither the glucose nor the lactate concentrations reached values which would inhibit cell proliferation.

In monolayer culture, MSCs are typically cultured on standard tissue culture plastic which is negatively charged (e.g. Nunclon Delta Surface and Corning CellBIND) but many culture dishes with positively charged surfaces are now available as this is believed to enhance cell attachment (e.g. Nunc and Corning Poly‐D‐lysine surfaces). Indeed, it has been demonstrated by Chun et al. that positively charged poly(lactic‐co‐glycolic acid) (PLGA) particles demonstrated greater levels of attachment and proliferation compared to negatively charged ones 39. This is in contrast to the data shown here and that of Kehoe et al. 24 where the neutrally charged Plastic and Collagen outperform their positively charged equivalents (Plastic Plus and FACT respectively, Table 1) with respect to cell growth. This emphasizes the complexity of the interaction between cells and material and that surface charge alone does not determine the capacity of a material to support cell attachment and/or growth. Indeed, surface charge density, hydrophobicity and topography have all been shown to impact cell adhesion, proliferation and even phenotype 40, 41, 42, 43.

Interestingly, in this study, the SoloHill microcarriers, in particular Plastic, Collagen, FACT and ProNectin F (Table 1), which all have the same polystyrene base but different coatings and charges, performed best with respect to cell growth in both the static/agitated conditions as well as the microwell plate studies. Notably, Cytodex‐3 and the SoloHill Collagen microcarriers which have the same biological coating but contain a different core material performed very differently. Our data therefore suggest that the fundamental nature of the particle (i.e. shape, size and density of the bead or mechanical stiffness of the base material) may also be a key factor in providing a conducive surface for cell adhesion and growth. Indeed, the stiffness of a surface matrix has previously been shown to affect MSC proliferation rate 42.

### 4.2 Significant improvement in hBM‐MSC yield in agitated cultures compared to static conditions

Of the major findings of this study, most notable is the effect of agitation on hBM‐MSC microcarrier culture (Fig. 3) and the wider implications this has on cell therapy bioprocessing. There is a significant difference when comparing the metabolite concentration and viable cell number for both the static and agitated cultures. More glucose is consumed and more lactate produced under agitated conditions in all cases, demonstrating that the cells are more metabolically active (and presumably more proliferative) in an agitated environment. This is not unexpected as it is widely acknowledged that agitation generates a homogenous physical and chemical environment by improving mass transfer and reducing concentration gradients 18. In addition to this, an agitated environment exposes the entirety of the microcarrier surface area to the cells, whereas static conditions do not. Indeed, one of the reasons to employ a microcarrier‐based culture is to attain the benefits of a well‐mixed, agitated system 21, 44.

Whilst the difference between static and agitated conditions for microcarrier culture is significant, it is unlikely that a microcarrier‐based process will ever be conducted under static conditions. What this does illustrate however is the importance of a well‐mixed system for improving cell yields. Even if a process is to be scaled via a monolayer expansion platform, efforts should be made to provide a mixed system and promote mass transfer. A prime example of this for monolayer culture are the roller bottle manufacturing platforms which are similar in principle to a T‐flask based process. However these have the added advantage of improved mass transfer via a rolling mechanism.

### 4.3 Key implications for cell therapy bioprocessing

A key finding from this study, and one that has potentially significant bioprocessing implications, is that irrespective of how the hBM‐MSCs from different donors compare with each other with respect to growth, there is a general trend as to microcarriers which appear to promote favorable growth. For example, despite the differences in growth kinetics between hBM‐MSC1, hBM‐MSC2 and hBM‐MSC3, they all performed better on Collagen, Plastic and ProNectin F in comparison to other microcarriers. It therefore suggests that once a microcarrier has been selected following a rigorous screening process as developed in this study, a process can be built around that particular microcarrier and there may not be the need to develop an entirely new process should the original donor cell bank be depleted and a new donor cell bank introduced. Productivity may be affected, but this is not due to the microcarrier and this can be minimized for an allogeneic process by sourcing donor cells similar to the original cells. Similarly, the fact that the donor cell growth kinetic performance relative to one another was maintained from monolayer through to agitated microcarrier culture suggests that donor cell screening for microcarrier culture needn't necessarily take place on microcarriers, but potentially, a relatively cheaper and more routine monolayer culture may suffice, thereby allowing for significant cost, resource and time savings. Finally, it is also worth noting that the relative performance of the microcarriers with the small‐scale, static microwell plate studies was identical to the findings in the larger‐scale agitated spinner flasks, again suggesting that a relatively cheaper and smaller microwell screening experiment may suffice as a high‐throughput microcarrier screening process, particularly when comparing the performance of multiple commercially available or in‐house produced microcarriers and when utilizing expensive media and reagents.

## 5 Concluding remarks

Employing the most appropriate microcarrier for hBM‐MSC expansion is a critical component of a microcarrier‐expansion process. This study developed a systematic method for selecting an appropriate microcarrier for the culture of hBM‐MSCs. Findings from this investigation suggest that whilst an agitated bioreactor comparison study would be definitive, microwell screening study may be sufficient for high‐throughput comparison of multiple microcarriers. Following an evaluation of thirteen commercially available microcarriers the SoloHill Plastic microcarrier was selected as the optimal microcarrier for hBM‐MSC expansion based on the following criteria: (i) extent of cell proliferation on the microcarrier; (ii) amenability for xeno‐free processing; and (iii) the ability to effectively harvest the cells from the microcarrier without any detrimental effect on cellular immunophenotype and differentiation capacity.

The relative performance of microcarriers was identical for each of the donor hBM‐MSCs, which has potentially significant implications for cell therapy bioprocessing as it would suggest that for an allogeneic process, a process will not need to be revalidated with a new microcarrier if a donor cell bank is exhausted. Similarly, it was demonstrated that agitated conditions were significantly better than static conditions, reinforcing the need for providing a homogenous culture environment irrespective of the culture platform.

## Supporting information

As a service to our authors and readers, this journal provides supporting information supplied by the authors. Such materials are peer reviewed and may be re‐organized for online delivery, but are not copy‐edited or typeset. Technical support issues arising from supporting information (other than missing files) should be addressed to the authors.

Supporting Information
