# Supplementary material for: Systematic microcarrier screening and agitated culture conditions improves human mesenchymal stem cell yield in bioreactors
Source: Biotechnol J. 2016 Feb 29;11(4):473–86. doi: 10.1002/biot.201400862 (PMC4991290; doi:10.1002/biot.201400862)
Supplement: Supplementary file 1 — Supporting Information [file BIOT-11-473-s001.pdf]

Biotechnology
Journal

Supporting Information for DOI 10.1002/biot.201400862

Systematic microcarrier screening and agitated culture conditions improves human mesenchymal stem cell yield in bioreactors

Qasim A. Rafiq, Karen Coopman, Alvin W. Nienow and Christopher J. Hewitt

© 2015 Wiley-VCH Verlag GmbH & Co. KGaA, Weinheim

Supporting information

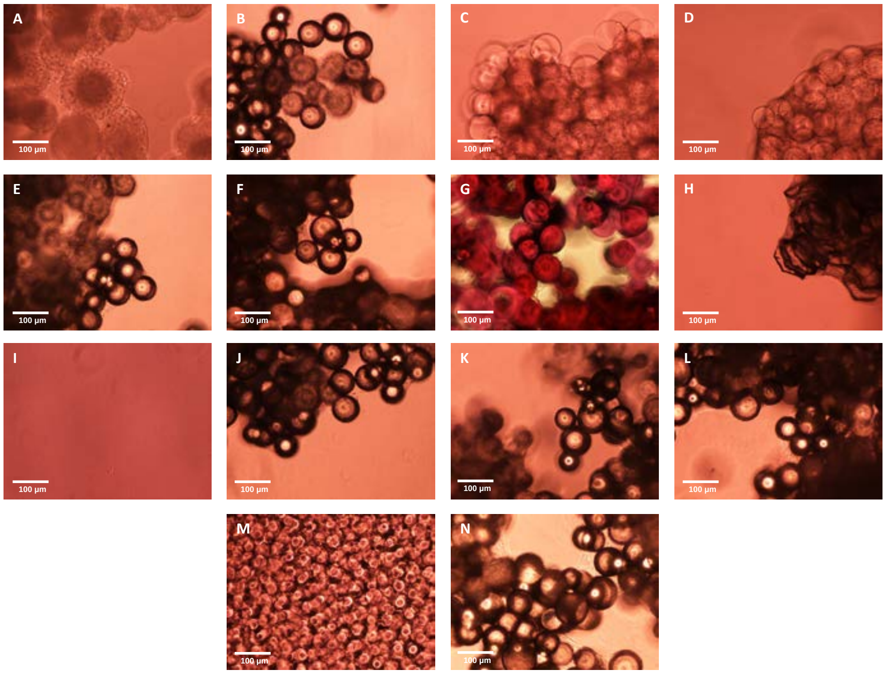

Figure S1: Phase contrast images of hBM-MSC1 donor cells attached to the different microcarriers in static microwell plates after 144 h. (A) Cultispher-G® (CG), (B) Collagen (Coll), (C) Cytodex-1TM (Cyto1), (D) Cytodex-3TM (Cyto3), (E) Enhanced Attachment (EA), (F) FACT, (G) Hillex®, (H) MicroHexTM (Mhex), (I) No microcarrier – control condition (No MC), (J) Plastic (Plas), (K) Plastic Plus (Pplus), (L) Pro-Nectin® F (Pro-F), (M) PVA, (N) Synthemax II® (Sy).

Osteogenic **Adipogenic** **Chondrogenic**

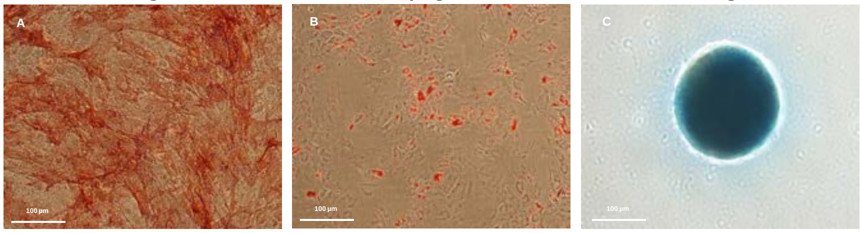

Alkaline Phosphatase and Von Kossa staining | Oil Red O staining | Alcian Blue staining

D

	Pre-inoculation (Day 0)	Post-harvest (Day 6)
**CD73 (+ve)**	98.9 ± 0.10	99.4 ± 0.15
**CD90 (+ve)**	99.9 ± 0.00	99.9 ± 0.00
**CD105 (+ve)**	99.3 ± 0.30	99.6 ± 0.05
**CD29 (+ve)**	98.3 ± 0.20	98.7 ± 0.05
**CD14 (-ve)**	0.39 ± 0.02	0.65 ± 0.08
**CD19 (-ve)**	0.77 ± 0.05	0.73 ± 0.11
**CD34 (-ve)**	0.22 ± 0.02	0.29 ± 0.00
**CD45 (-ve)**	0.83 ± 0.03	0.66 ± 0.15
**HLA-DR (-ve)**	0.67 ± 0.11	1.21 ± 0.09

Figure S2: Cell characterisation data for hBM-MSC1 harvested from the Plastic microcarriers after spinner flask culture. Demonstration of differentiation towards osteogenic (A), adipogenic (B) and chondrogenic (C) lineages. Expression of positive cell (CD73, CD90, CD105 and CD29) and negative (CD14, CD19, CD34, CD45 and HLA-DR) surface markers (D). Data is presented as mean ± SD (n=4).
